# Numerical investigation for rotating flow of MHD hybrid nanofluid with thermal radiation over a stretching sheet

**DOI:** 10.1038/s41598-020-75254-8

**Published:** 2020-10-28

**Authors:** Muhammad Shoaib, Muhammad Asif Zahoor Raja, Muhammad Touseef Sabir, Saeed Islam, Zahir Shah, Poom Kumam, Hussam Alrabaiah

**Affiliations:** 1 Department of Mathematics, COMSATS University Islamabad, Attock Campus, Islamabad, Pakistan; 2 Future Technology Research Center, National Yunlin University of Science and Technology, 123 University Road, Section .3, Douliou, Yunlin, 64002 Taiwan, ROC; 3 Department of Electrical and Computer Engineering, COMSATS University Islamabad, Attock Campus, Islamabad, Pakistan; 4 Faculty of Mathematics and Statistics, Ton Duc Thang University, Ho Chi Minh City, 70000 Vietnam; 5 Informetrics Research Group, Ton Duc Thang University, Ho Chi Minh City, 70000 Vietnam; 6 Department of Mathematics, University of Lakki Marwat, Lakki Marwat, 28420 Khyber Pakhtunkhwa Pakistan; 7 KMUTT Fixed Point Research Laboratory, Room SCL 802 Fixed Point Laboratory, Science Laboratory Building, Department of Mathematics, Faculty of Science, King Mongkut’s University of Technology Thonburi (KMUTT), Bangkok, 10140 Thailand; 8 Department of Medical Research, China Medical University Hospital, China Medical University, Taichung, 40402 Taiwan, ROC; 9 College of Engineering, Al Ain University, Al Ain, 64141 UAE; 10 Department of Mathematics, Tafila Technical University, Tafila, 66110 Jordan

**Keywords:** Mathematics and computing, Fluid dynamics, Mechanical engineering

## Abstract

This research investigates the heat and mass transfer in 3-D MHD radiative flow of water based hybrid nanofluid over an extending sheet by employing the strength of numerical computing based Lobatto IIIA method. Nanoparticles of aluminum oxide (Al_2_O_3_) and silver (Ag) are being used with water (H_2_O) as base fluid. By considering the heat transfer phenomenon due to thermal radiation effects. The physical flow problem is then modeled into set of PDEs, which are then transmuted into equivalent set of nonlinear ODEs by utilizing the appropriate similarity transformations. The system of ODEs is solved by the computational strength of Lobatto IIIA method to get the various graphical and numerical results for analyzing the impact of various physical constraints on velocity and thermal profiles. Additionally, the heat transfers and skin friction analysis for the fluid flow dynamics is also investigated. The relative errors up to the accuracy level of 1e-15, established the worth and reliability of the computational technique. It is observed that heat transfer rate increases with the increase in magnetic effect, Biot number and rotation parameter.

## Introduction

Suspension of uniformly dispersed and stable nanoparticles in base fluid e.g. water (H_2_O), methanol and ethylene etc. are called nanofluids. Properties of every nanofluid vary from other nanofluid depending upon these tiny particles and base fluid being used. These nanoparticles comprise of metals, oxides of metals like Ag, Cu, TiO_2_, SiO_2_, Fe_2_O_3_ and carbon nanotubes (SWCNTs and MWCNTs) etc. The average diameter of these suspended nanoparticles is of order less the nanometer (nm). Low thermal conductivity of ordinary base fluids like water and ethylene limit their role for being used separately in several practical fields. To overcome the disadvantage of low thermal capability of these conventional base fluids, a modern type of fluid recognized as nanofluids are introduced by enhancing their thermal characteristics with the use of various kind of nanoparticles^1^. Due to the numerous heat transmission properties, this class of fluids is excessively used in industrial and engineering applications such as cooling of electronic equipment, cooling process in HVAC systems, refrigeration processes, food industry, solar collectors and micro channel heat sink etc.^2–5^. Solar collectors are the devices that convert the solar energy into heat energy in eco-friendly and convenient way. Several type of nanofluids are used in these solar collector for improving their ability to convert the requisite form of energy. Utilization of nanofluid for the enhancement of thermal capabilities of such solar collectors are numerically and experimentally studies by many researchers.

Idea of nanofluid was first time experimentally presented by Choi et al.^6^ in 1995 and observed the enhancement of thermal efficiency as compared to simple base fluid. These results were then experimentally verified by Kang et al.^7^. Later on, the thermal conductivity of nanofluid based on water comprising the nanoparticles of copper (Cu) and aluminum oxide (Al_2_O_3_) was measured by Eastman et al.^8^ and Lee et al.^9^. Enhancement in thermal conductivity can unswervingly improve the rate of transfer of heat capabilities of nanofluids comparatively if it is compared with ordinary base fluids. Keeping in view, the idea of thermal conductivity of nanofluids, various type of flow models along with displaying procedures and applications of hybrid nanofluids have been discussed^10^. Ahmed et al.^11^ evaluated the squeezing flow dynamics of nanofluid comprising Al_2_O_3_ nanoparticles between two parallel disks. Additionally, numerical and analytical results of heat transfer and skin friction were also highlighted. Sun et al.^12^ studied experimentally the variation of heat transfer rate for nanofluid (Fe_3_O_4_/H_2_O) inside the horizontal circular tubes under the effects of magnetic field and found a direct relationship between magnetic field strength and rate of heat transfer. Kumar et al.^13^ numerically compared the transfer rate of heat for ordinary fluid and nanofluid (Al_2_O_3_/H_2_O) systems, and declared that with the use of nanofluid, decrease in temperature, thermal resistance and power consumption is observed whereas, the reliability of the electronic chips increases by 70% with the use of nanofluid. Lahmar et al.^14^ inspected the behavior of thermal conductivity and heat transfer rate in squeezing flow of Fe_3_O_4_/H_2_O inside two parallel plates with the effect of magnetic field. Asha et al.^15^ analyzed the peristaltic blood flow with nanoparticles of gold (Au) inside an irregular channel and discussed the hall current effects on flow. Gbadeyan et al.^16^ discussed the MHD flow of Casson nanofluid over a convectively heated vertical plate with velocity slip effects. Additionally, impact of thermal conductivity and radiation phenomenon on the flow is also presented graphically and numerically. Some details about heat transfer in various fluidic systems including respective nanoparticles are shown in Table 1.

**Table 1:** Improvement in heat transfer of fluids by using various nanoparticles.

Base fluid	Nano particle	Increase in conductance	Volume concentration	References
Water (H_2_O)	TiO_2_	7.4%	0.2–3.0%	Turgut et al.^17^
Water (H_2_O)	CuO	34%	0.0–16%	Mintsa et al.^18^
Water (H_2_O)	Al_2_O_3_	31%	0.0–18%	
Water (H_2_O)	Al_2_Cu	76%	1.0–2.0%	Chopkar et al.^19^
Water (H_2_O)	Ag_2_Al	93%	1.0–2.0%	
Water (H_2_O)	SiC	24%	1.0–4.0%	Xie et al.^20^
Ethylene glycol (EG)	MWCNT	30%	1.0–2.0%	Liu et al.^21^
Ethylene glycol (EG)	Al_2_O_3_	19%	2.0–3.0%	Beck et al.^22^
Ethylene glycol (EG)	Fe	18%	0.10–0.55%	Hong et al.^23^

A remarkable volume of studies has been carried out on manufacturing, classification and applications of different types of nanofluids. But hybrid nanofluid are modern sort of nanofluids, which are manufactured by two or more than two kinds of nanoparticles either in mixture or compound form. The purpose for this process is to achieve the best possible combination of chemical and physical properties of different materials simultaneously in a unique fluid. Synthetic hybrid material shows extraordinary chemical and physical properties which cannot be attained through any of component in individual state. Hybrid nanofluids are new and innovative type of fluids and judgment of their performance is still under evaluation phase. In recent past years, few researches have been conducted for the comparison of performance between nanofluid and hybrid nanofluids^24–27^.

Magnetohydrodynamics (MHD) is the study where the magnetic field and the velocity field are coupled, given there is an electrically conducting fluid. The magnetic field can induce currents into such a moving fluid and this creates forces acting on the fluid and altering the magnetic field itself. Set of differential equations comprises of Navier–Stokes equations and Maxwell’s equations describes the complete phenomenon of MHD. Kashi’ie et al.^28^ numerically investigated the flow properties for the dynamics of fluidic system and phenomena of heat transfer for a MHD flow of hybrid nanofluid (Al_2_O_3_/H_2_O) due to stretching sheet while considering the joule heat effects. Osho et al.^29^ discovered the flow characteristics of hybrid nanofluid (Al_2_O_3_-Zn/H_2_O) and noticed the significant effect of concentration of nanoparticles over the viscosity and specific heat of the flow. Aly et al.^30^ theoretically and numerically studied the MHD stagnation point flow over stretching sheet of hybrid nanofluid with dissipation and slip effects and observed a relationship between MHD and rate of heat transfer. Aghahadi et al.^31^ inspected the rheological performance of tungsten oxide-engine oil nanofluid at various concentration and temperature and found a linear relationship between applied shear stress and shear rate. Nagoor et al.^32^ numerically explicated the influence of various physical constraints on velocity and temperature fields for Darcy-Forchheimer hybrid nanofluid in rotating frame by using Lobatto IIIA method. Huminic et al.^33, 34^ discussed heat transfer rate and entropy generation between ordinary and hybrid nanofluid in different physical situations. Saba et al.^35^ numerically explored the phenomena of heat transfer for a hybrid nanofluid in an irregular channel with permeable walls. Furthermore, various effective results have been illustrated via plots. Oliverira et al.^36^ experimentally studied an innovative method for addition of silver on the surface of diamond nanoparticle for the preparation of hybrid nanoparticles (Di-Ag). Different techniques including scanning electron microscopy (SEM) as well as X-ray diffraction (XRD) are executed to get required information about these hybrid nanoparticles. Lund et al.^37^ examined the influence of different factors on the velocity and temperature profiles of a hybrid nanofluid (Cu–Al_2_O_3_/H_2_O) over stretched sheet under the effects of suction and viscous dissipation. Shahsavar et al.^38^ inspected the impacts of concentration on entropy generation and heat transfer of non-Newtonian iron oxide-based hybrid nanofluid through concentric annulus. Iqbal et al.^39^ inspected the Hall current effects on MHD flow of hybrid nanofluid in revolving channel under thermal radiations with different shapes of nanoparticles. During the recent past, many researchers investigated the heat transfer phenomenon in nanofluid flow^40–48^.

The inspiration behind this research work is above referred studies in which several researchers assumed various fluid with different types of nanoparticles and observed fascinating results for their thermal properties. A considerable research is being done about the numerical solution of the nanofluid flow problem^49–51^, but very few researchers tried to solve the hybrid nanofluid flow problem with novel numerical techniques. In this article, the authors investigate the problem of 3-D flow of MHD hybrid nanofluid over an extendable sheet in presence of thermal radiation. Main features of this study are as follows:

- A novel scheme for 3-D MHD flow of hybrid nanofluid over an extendable sheet with thermal radiation effects has been modeled. System of PDEs expressing the flow model is then transmuted into the set of equivalent nonlinear ODEs while employing the appropriate mathematical transformations.
- Detailed numerical study of the flow model is described by implementing the computational strength of Lobatto IIIA method with the aim to scan the influence of involved physical constraints on velocity and thermal fields.
- To achieve the required solution of highly nonlinear ODEs, use of Lobatto IIIA technique in MATLAB software for this problem is an inventive work. Lobatto IIIA is the kind of bvp4c scheme depends on FDM. The strength of this technique is to solve the higher order nonlinear ODEs.
- Detailed graphical and numerical explanation of result has also been presented, which evidently shows the variation of velocity and thermal fields on several constraints of interest.

## Problem formulation

**Figure 1:**
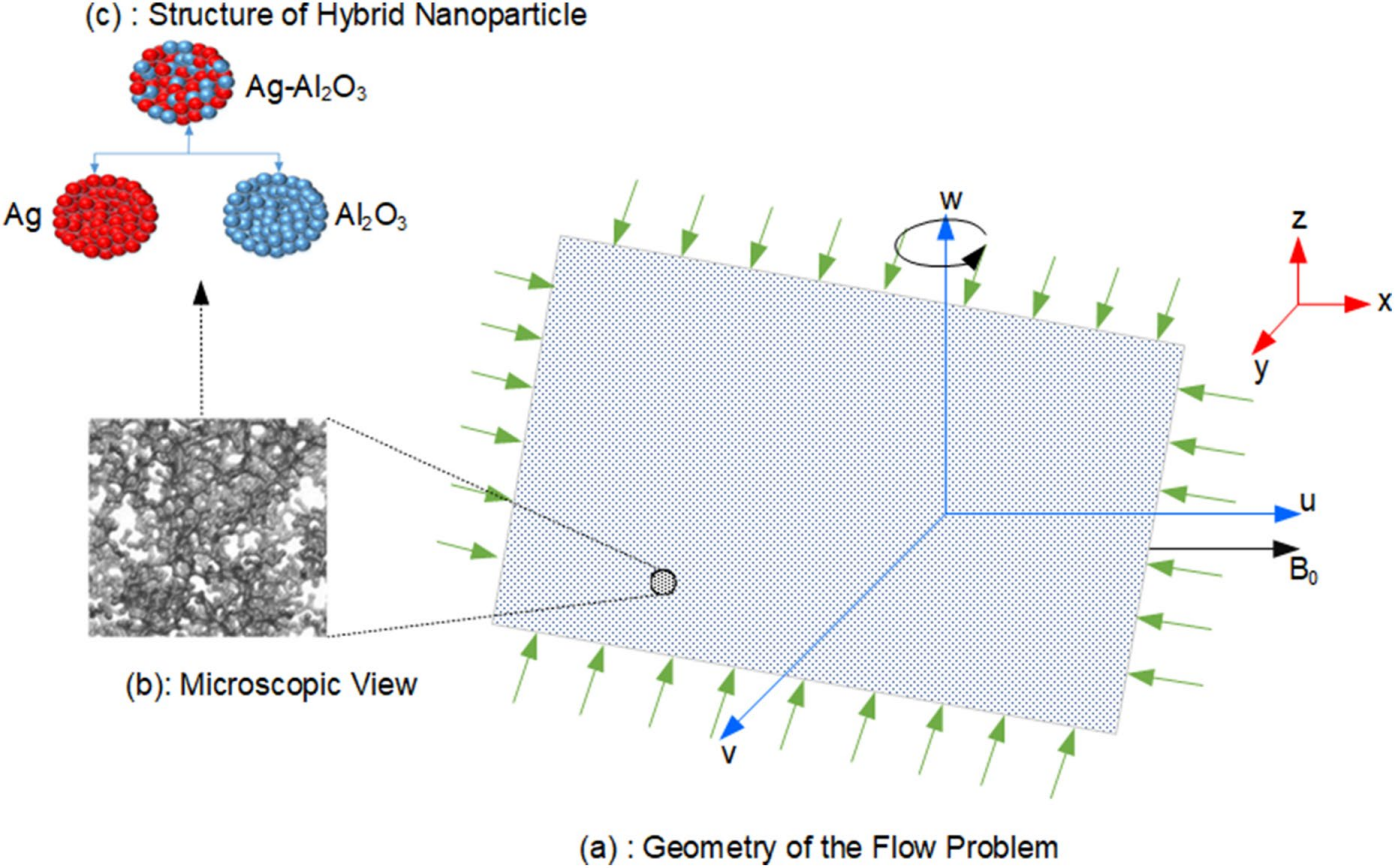
Flow diagram of the problem.

Consider the incompressible 3-D flow of hybrid nanofluid induced by a stretching and rotating effects with thermal convection and radiation along a sheet. The sheet is stretched through selected xy-coordinates system and nanofluid is assumed for $z > 0$ direction. Velocity components in $x,y\;{\text{and}}\;z$ direction are denoted by $u, v\;{\text{and}}\;w$, respectively. Figure 1 displays the schematic view of flow model in which Fig. 1a presents the geometry of the problem, Fig. 1b shows the microscopic view of surface and Fig. 1c depicts the structure of hybrid nanoparticles. $T_{f}$ and $T$ denote the surface and fluid temperatures respectively, while the applied constant magnetic field acting in parallel direction to z-axis is represented by B_0_ and $h_{f}$ is the coefficient of heat transfer.

Hence, the balance of mass, balance of momentum and energy can be mathematically expressed as^52, 53^:

1 $$\frac{\partial u}{{\partial x}} + \frac{\partial v}{{\partial y}} + \frac{\partial w}{{\partial z}} = 0,$$

2 $$\left[ {u\frac{\partial u}{{\partial x}} + v\frac{\partial u}{{\partial y}} + w\frac{\partial u}{{\partial z}} - 2\omega v} \right] = \nu_{hnf} \left[ {\frac{{\partial^{2} u}}{{\partial z^{2} }}} \right] - \sigma_{hnf} B_{0}^{2} u,$$

3 $$\left[ {u\frac{\partial v}{{\partial x}} + v\frac{\partial v}{{\partial y}} + w\frac{\partial v}{{\partial z}} + 2\omega u} \right] = \nu_{hnf} \left[ {\frac{{\partial^{2} v}}{{\partial z^{2} }}} \right] - \sigma_{hnf} B_{0}^{2} v,$$

4 $$\left( {\rho c_{p} } \right)_{hnf} \left[ {u\frac{\partial T}{{\partial x}} + v\frac{\partial T}{{\partial y}} + w\frac{\partial T}{{\partial z}}} \right] = \left( {k_{hnf} + \frac{16}{3}\frac{{\sigma^{*} T_{\infty }^{3} }}{{k^{*} }}} \right)\left[ {\frac{{\partial^{2} T}}{{\partial z^{2} }}} \right],$$

Corresponding boundary condition are:

5 $$\begin{aligned} & u = u_{w} = bx,\;v = 0,\;w = 0, - k_{hnf} \left( {\frac{\partial T}{{\partial z}}} \right) = h_{f} \left( {T_{f} - T} \right)\;at\;z = 0, \\ & u \to 0,\;v \to 0,\;T \to T_{\infty } \;at\;z \to \infty . \\ \end{aligned}$$

Mathematical relationships for various Thermophysical characteristics for hybrid nanofluids are^54^:

$$\mu_{hnf} = \frac{{\mu_{f} }}{{\left[ {\left( {1 - \phi_{1} } \right)\left( {1 - \phi_{2} } \right)} \right]^{2.5} }},\; \rho_{hnf} = \rho_{f} \left[ {\left( {\frac{{\rho_{s1} }}{{\rho_{f} }}} \right)\phi_{1} + \left( {1 + \phi_{1} } \right)} \right]\left( {1 - \phi_{2} } \right) + \phi_{2} \rho_{s2} ,$$

$$\left[ {\rho c_{p} } \right]_{hnf} = \left[ {\rho c_{p} } \right]_{f} \left[ {\left( {1 - \phi_{1} } \right) + \frac{{\left( {\rho c_{p} } \right)_{s1} }}{{\left( {\rho c_{p} } \right)_{f} }}\phi_{1} } \right]\left( {1 - \phi_{2} } \right) + \phi_{2} \left( {\rho c_{p} } \right)_{s2} ,$$

$$\frac{{k_{hnf} }}{{k_{bf} }} = \frac{{k_{s2} + k_{bf} \left( {s - 1} \right) - \left( {k_{bf} - k_{s2} } \right)\left( {s - 1} \right)\phi_{2} }}{{\left( {k_{bf} - k_{s2} } \right)\phi_{2} + \left( {s - 1} \right)k_{bf} + k_{s2} }},$$

$$\frac{{k_{bf} }}{{k_{f} }} = \frac{{k_{s1} + k_{f} \left( {s - 1} \right) - \left( {k_{f} - k_{s1} } \right)\left( {s - 1} \right)\phi_{1} }}{{k_{f} \left( {s - 1} \right) + \left( {k_{f} - k_{s1} } \right)\phi_{1} + k_{s1} }}.$$

**Figure 2:**
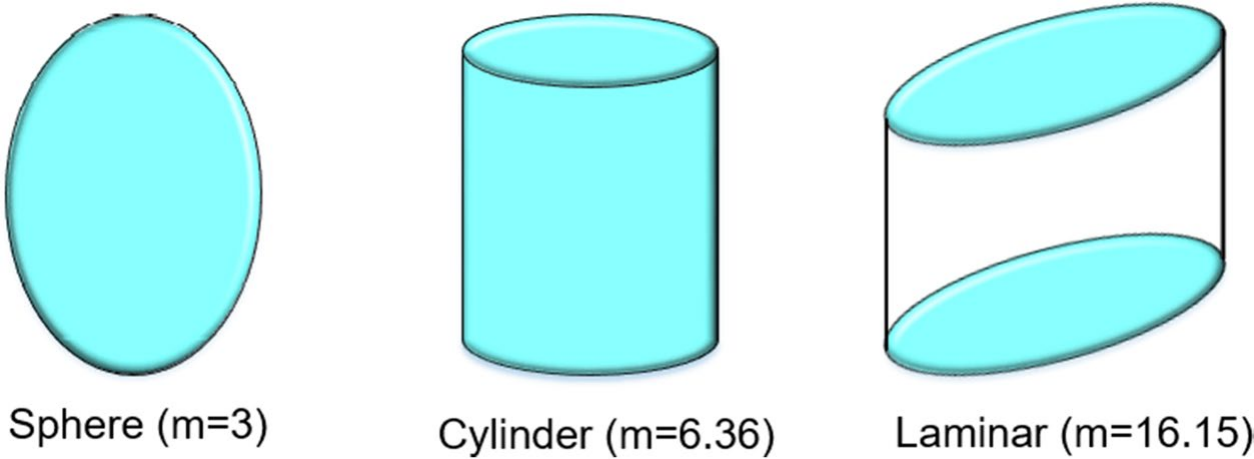
Geometrical appearance (size) of nano particles^55^.

The hybrid nanofluid consists of mixtures of Al_2_O_3_ and Ag nanoparticles in base fluid water (H_2_O). The concentration of Al_2_O_3_ and Ag nanoparticles are denoted by $\phi_{1}$ and $\phi_{2}$ respectively, whereas $\phi_{hnf}$ is the total concentration of mix nanoparticles which can simply be calculated as $(\phi_{1} + \phi_{2} )$. Values for density, thermal conductivity and specific heat of base fluids and nanoparticles are placed in Table 2, whereas Fig. 2 displays the well-known shapes of nanoparticles with numerical values of size, while $\rho_{f} , \rho_{s1} , \rho_{s2}$ represent the density of fluid, Al_2_O_3_ particles and Ag particles, respectively. Thermal conductivity of Al_2_O_3_ particles, Ag particles, base fluid and hybrid nanofluid is represented by $k_{s1} ,k_{s2} ,k_{f} \;and\;k_{hnf}$, respectively, $\left( {C_{p} } \right)_{s1} ,\left( {C_{p} } \right)_{s2} ,\left( {C_{p} } \right)_{f} \;and\;\left( {C_{p} } \right)_{hnf}$ represent the specific heat of Al_2_O_3_ particles, Ag particles, base fluid and hybrid fluid, respectively.

**Table 2:** Numerical values of various properties for fluids and nanoparticles^54–56^.

Properties	Base fluids			Nano particles			
	H_2_O	Kerosene	Engine oil	Cu	Al_2_O_3_	Ag	SiO_2_
Density ($\rho$) (kg m^−3^)	997.1	783	884	8933	3970	10,500	2200
Thermal conductivity (k) (W m^-1^ K^−1^)	0.613	0.145	0.144	400	40	429	1.4
Specific heat (Cp) (J kg^−1^ K^−1^)	4179	2090	1910	385	765	235	703

Dimensional form for coefficient of skin friction and Nusselt number can be written as^57^:

6 $$C_{f} = \frac{{\mu_{hnf} }}{{\rho_{f} u_{w}^{2} }}\left( {\frac{\partial u}{{\partial z}}} \right)_{z = 0} ,\;C_{g} = \frac{{\mu_{hnf} }}{{\rho_{f} u_{w}^{2} }}\left( {\frac{\partial v}{{\partial z}}} \right)_{z = 0} ,\;Nu = - \frac{{xk_{hnf} }}{{k_{f} \left( {T - T_{\infty } } \right)}}\left( {\frac{\partial T}{{\partial z}}} \right)_{z = 0} .$$

To reduce the system of PDEs (1–4) into dimensionless set of ODEs, following mathematical transformations are introduced:

7 $$\begin{aligned} & u = bxf^{\prime}\left( \eta \right),v = bxg\left( \eta \right),w = - \sqrt {b\nu _{f} } f\left( \eta \right), \\ & \eta = \sqrt {\frac{b}{{\nu _{f} }}~} z,\theta \left( \eta \right) = \frac{{T - T_{\infty } }}{{T_{f} - T_{\infty } }}. \\ \end{aligned}$$

Substituting above-mentioned transformations, the continuity equation is identically satisfied, while the Eqs. (2–4) take the following form:

8 $$f^{{{\prime \prime \prime }}} - A_{1} A_{2} \left[ {f^{{{\prime }2}} - f^{{\prime \prime }} f - 2\Omega g + Mf^{{\prime }} } \right] = 0,$$

9 $$g^{{\prime \prime }} - A_{1} A_{2} \left[ {f^{{\prime }} g - fg^{{\prime }} - 2\Omega f^{{\prime }} + Mg} \right] = 0,$$

10 $$\left[ {\frac{{K_{hnf} }}{{K_{f} }} + \frac{4}{3}Rd} \right]\theta^{{\prime \prime }} + A_{3} \Pr \;f\theta^{{\prime }} = 0,$$

whereas,

11 $$\begin{aligned} & A_{1} = \left[ {\left( {1 - \phi _{1} } \right)\left( {1 - \phi _{2} } \right)} \right]^{{5/2}} ~,~A_{2} = \left[ {\phi _{1} \left( {\frac{{\rho _{{s1}} }}{{\rho _{f} }}} \right) + \left( {1 - \phi _{1} } \right)} \right]\left[ {1 - \phi _{2} } \right] + \left( {\frac{{\rho _{{s2}} }}{{\rho _{f} }}} \right)~\phi _{2} , \\ & A_{3} = \left[ {1 - \phi _{2} } \right]\left[ {\left( {\frac{{\left( {\rho c_{p} } \right)_{{s1}} }}{{\left( {\rho c_{p} } \right)_{f} }}} \right)\phi _{1} + \left( {1 - \phi _{1} } \right)} \right] + \left( {\frac{{\left( {\rho c_{p} } \right)_{{s2}} }}{{\left( {\rho c_{p} } \right)_{f} }}} \right)\phi _{2} . \\ \end{aligned}$$

The BC’s are

12 $$\begin{aligned} & f\left( \eta \right) = 0,\;g\left( \eta \right) = 0,\;f^{{\prime }} \left( \eta \right) = 1,\theta ^{{\prime }} \left( \eta \right) = - \frac{{K_{f} }}{{K_{{hnf}} }}\gamma \left( {1 - \theta \left( \eta \right)} \right)\;{\text{at}}\;\eta = 0, \\ & f^{{\prime }} \left( \eta \right) \to 0,~\;g\left( \eta \right) \to 0,\;\theta \left( \eta \right) \to 0\quad {\text{when}}\;\eta \to \infty . \\ \end{aligned}$$

Dimensionless parameters involved in Eqs. (8–12) can be written as:

13 $$\Omega = \frac{\omega }{b},\;Rd = \frac{{4\sigma T_{\infty }^{3} }}{{k^{*} k_{f} }},\;\Pr = \frac{{\upsilon_{f} \left( {\rho c_{p} } \right)_{f} }}{{k_{f} }},\;{\text{M}} = \frac{{\sigma_{nf} B_{0}^{2} }}{b},\;\gamma = \frac{{h_{f} }}{{k_{f} }}\sqrt {\frac{{\nu_{f} }}{b}}$$

Coefficient of Skin friction and Nusselt number in (6) are reduced to their dimensionless form as:

14 $$\begin{aligned} & C_{f} Re_{x} ^{{1/2}} = \frac{1}{{\left( {1 - \phi _{1} } \right)^{{5/2}} \left( {1 - \phi _{2} } \right)^{{5/2}} }}f^{{\prime\prime }} \left( 0 \right),\;C_{g} Re_{x} ^{{1/2}} = \frac{1}{{\left( {1 - \phi _{1} } \right)^{{5/2}} \left( {1 - \phi _{2} } \right)^{{5/2}} }}g^{{\prime }} \left( 0 \right), \\ & Nu\text{Re} _{x} ^{{ - 1/2}} = - \frac{{k_{{hnf}} }}{{k_{f} }}\theta ^{{\prime }} \left( 0 \right). \\ \end{aligned}$$

In which $Re_{x} = \frac{{u_{w} x}}{{\nu_{f} }}$. represents the Reynolds number.

## Solution methodology

**Figure 3:**
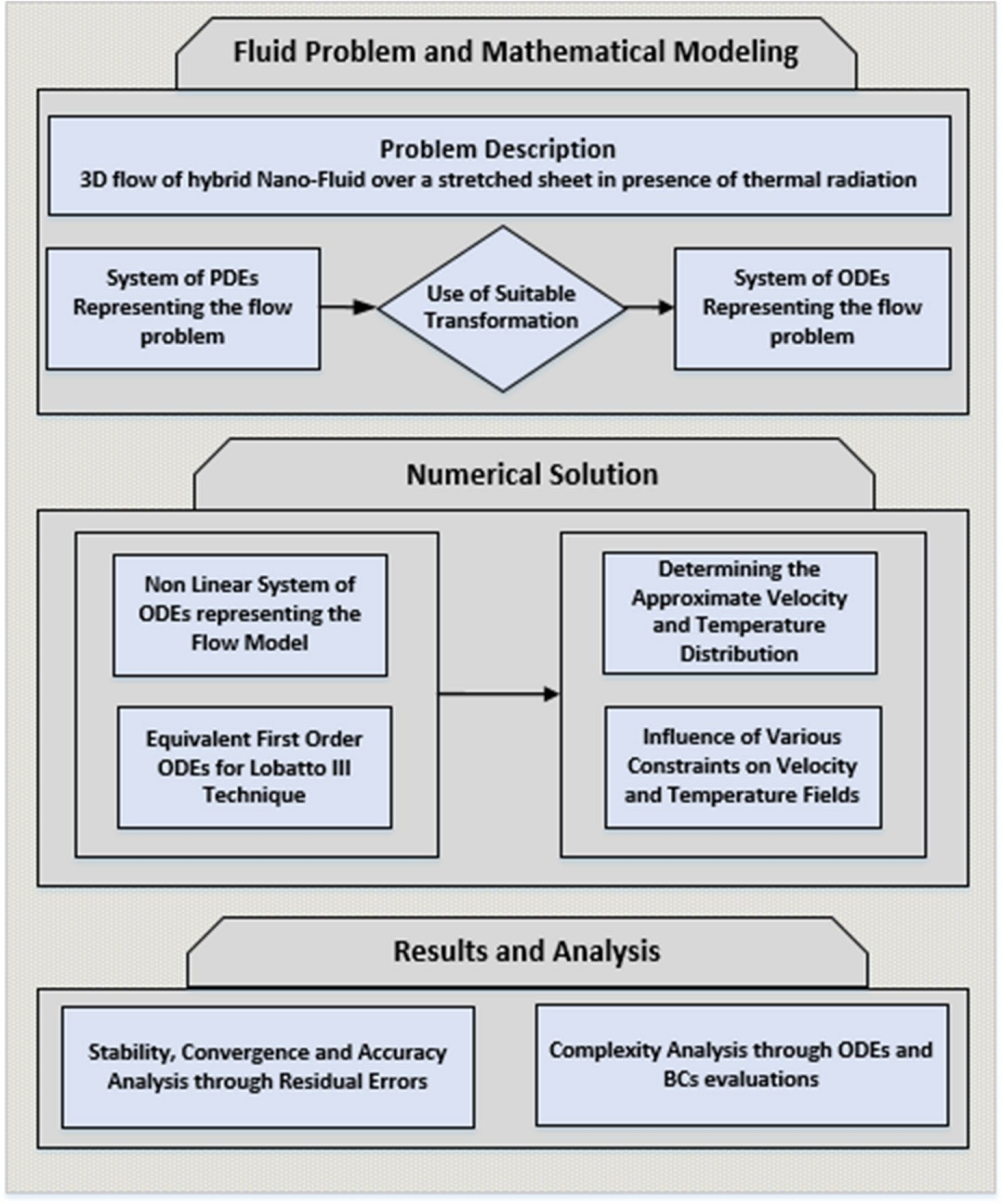
Working flow chart.

Transformed set of ODEs representing the flow problems given in Eqs. (8)–(12) are solved numerically by employing Lobatto IIIA technique in MATLAB software using bvp4c package as described in Fig. 3, while the detail information regarding the solution technique is available in^58, 59^. The obtained graphical and numerical results portray the impact of all involved parameters on velocity as well as temperature fields. The convergence, stability and accuracy have been checked for solution and computation with the help of residual error for each case of all scenarios. Equations (8)–(12) are transformed to first order system of ODEs by the Lobatto IIIA technique.

15 $$f_{1} = f^{{\prime }} ,\;f_{2} = f_{1}^{{\prime }} ,\;f_{3} = f_{2}^{{\prime }} = A_{1} A_{2} \left[ {f^{{{\prime }2}} - ff^{{\prime \prime }} - 2\Omega g + Mf^{{\prime }} } \right],$$

16 $$g_{1} = g^{{\prime }} ,\;g_{2} = f_{1}^{{\prime }} = A_{1} A_{2} \left[ {f^{{\prime }} g - fg^{{\prime }} - 2\Omega f^{{\prime }} + Mg} \right]$$

17 $$\theta_{1} = \theta^{{\prime }} ,\;\theta_{2} = \theta_{1}^{{\prime }} = \frac{{A_{3} Pr\;f\theta^{{\prime }} }}{{\frac{{k_{nf} }}{{k_{f} }} + \frac{4Rd}{3}}}$$

With the boundary conditions

18 $$\begin{aligned} & f = 0,\;f_{1} = 1,\;g = 0,\;\theta_{1} = \gamma \left( {1 - \theta } \right)\quad at\quad \eta = 0 \\ & f_{1} \to 0,\;g \to 0,\;\theta \to 0 \quad at\quad \eta \to \infty . \\ \end{aligned}$$

## Results and discussion

**Figure 4:**
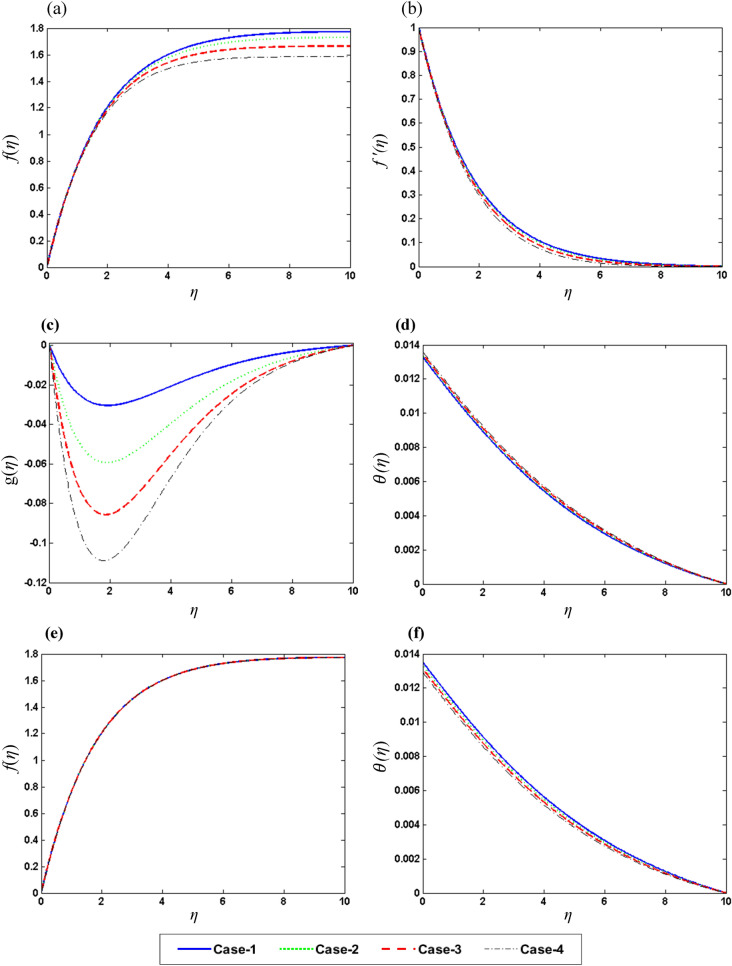
Graphic variation of (**a**) $f\left( \eta \right)$, (**b**) $f^{{\prime }} \left( \eta \right)$, (**c**) $g\left( \eta \right)$, (**d**) $\theta \left( \eta \right)$ on $\Omega$ (**e**) $f\left( \eta \right)$, (**f**) $\user2{ }\theta \left( \eta \right)$ on Pr.

**Figure 5:**
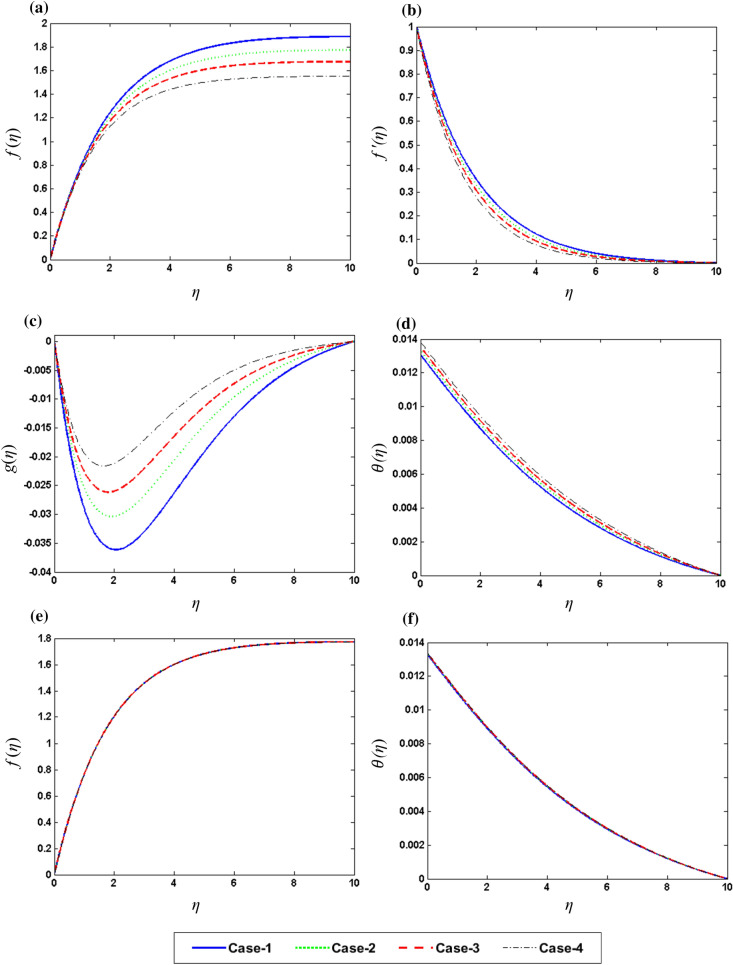
Graphic variation of (**a**) $f\left( \eta \right)$, (**b**) $f^{{\prime }} \left( \eta \right)$, (**c**) $g\left( \eta \right)$, (**d**) $\theta \left( \eta \right)$ on M (**e**) $f\left( \eta \right)$, (**f**) $\user2{ }\theta \left( \eta \right)$ on Rd.

**Figure 6:**
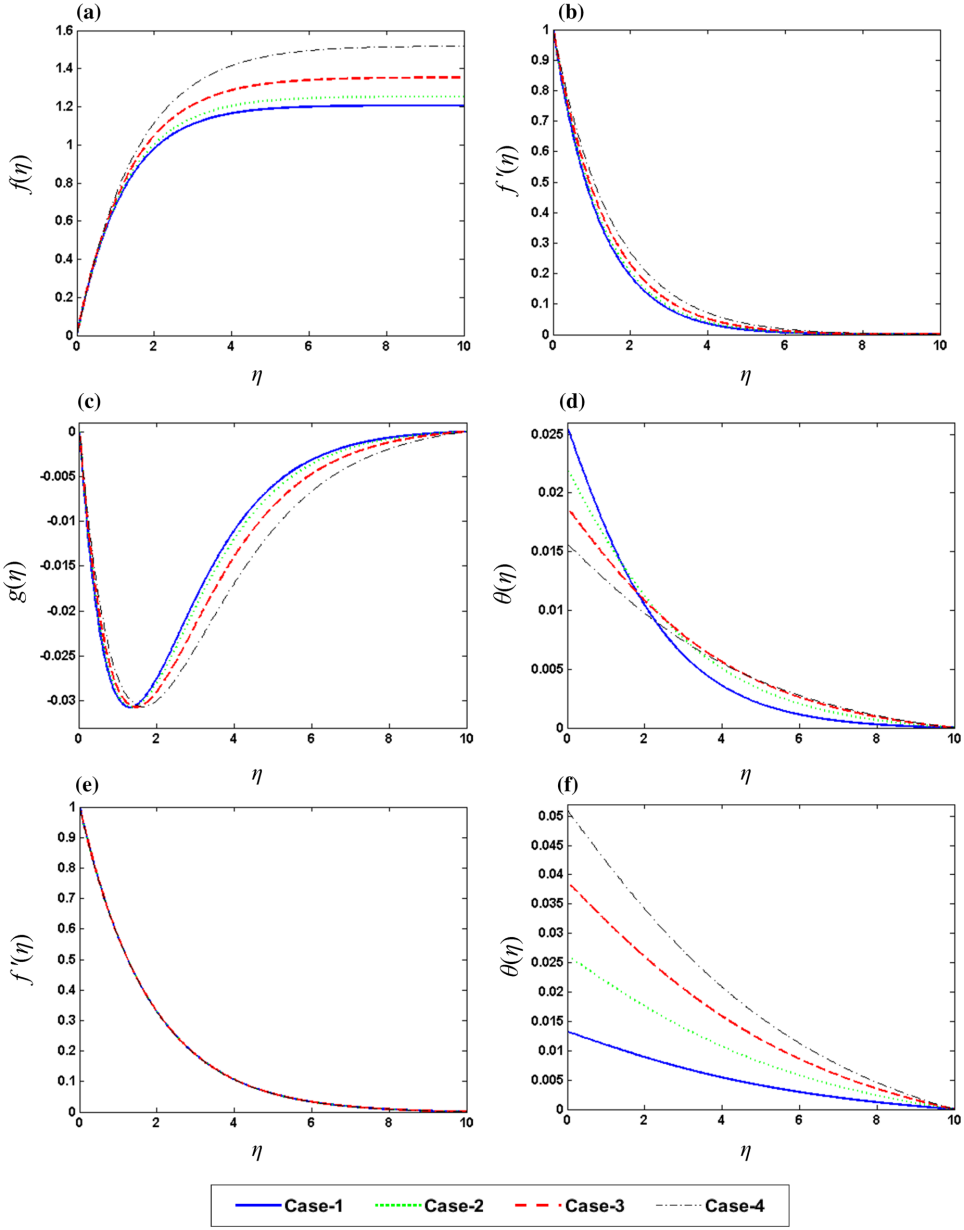
Graphic variation of (**a**) $f\left( \eta \right)$, (**b**)$f^{{\prime }} \left( \eta \right)$, (**c**) $g\left( \eta \right)$, (**d**) $\theta \left( \eta \right)$ on $\phi_{2}$ (**e**) $f^{{\prime }} \left( \eta \right)$, (**f**)$\user2{ }\theta \left( \eta \right)$ on $\gamma$.

**Figure 7:**
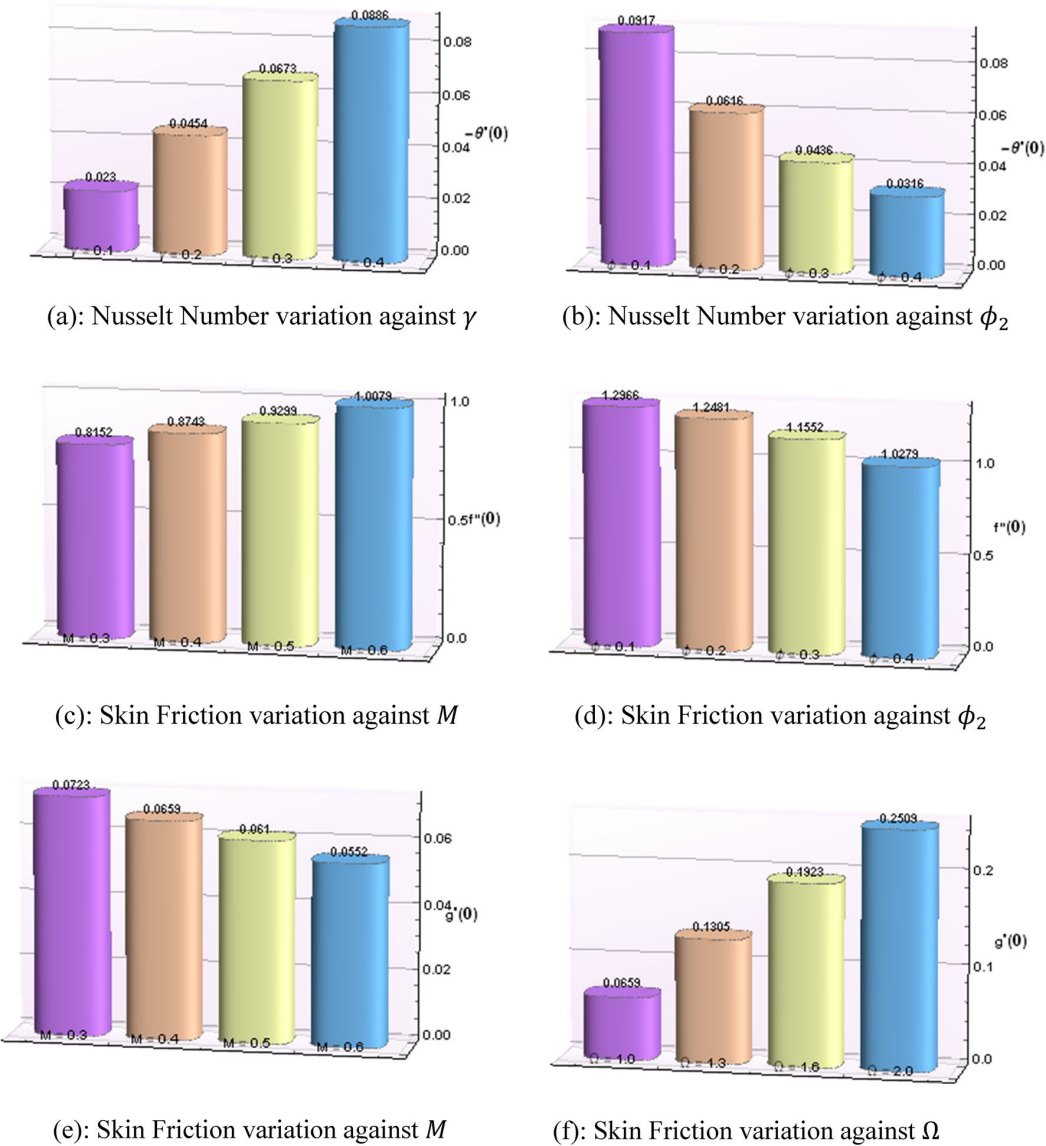
Values of skin friction and Nusselt number against different variable parameters.

After solving the resultant set of ODEs, various forms of numerical with its graphical outcomes are obtained and displayed in Figs. 4, 5, 6 and 7 and Tables 4, 5 and 6 to check the influence of all involved parameters of interest Rd (Radiation parameter), ${\Omega }$ (Rotation parameter), $\gamma \left( {{\text{Biot}}\;{\text{Number}}} \right)$, Pr (Prandtl number) , M (Magnetic parameter), and $\phi_{2}$ (Concentration of nanoparticles) on the velocity field $f\left( \eta \right),f^{{\prime }} \left( \eta \right),g\left( \eta \right)$ and temperature field $\theta \left( \eta \right)$. Six different scenarios of the system m presented in Eqs. 8–12 are formulated by variation in the values of $Rd,\;M,\;\gamma ,\;\Omega ,\;{\Pr}\;and\;\phi_{2}$ as shown in Table 3 to analyze the dynamic. Numerical simulation is performed for each scenario with four cases and observe their impact on the flow dynamics throughout in the presented study.

**Table 3:** Variation in values of involved physical parameters.

Scen	Case (I)	Case (II)	Case (III)	Case (IV)
1	$\Omega$ = 1.0	$\Omega$ = 1.3	$\Omega$ = 1.6	$\Omega$ = 2.0
2	Rd = 0.1	Rd = 0.3	Rd = 0.5	Rd = 0.7
3	M = 0.3	M = 0.4	M = 0.5	M = 0.6
4	$\gamma$ = 0.1	$\gamma$ = 0.2	$\gamma$ = 0.3	$\gamma$ = 0.4
5	Pr = 6.0	Pr = 6.2	Pr = 6.4	Pr = 6.6
6	$\phi_{2}$ = 0.1	$\phi_{2}$ = 0.2	$\phi_{2}$ = 0.3	$\phi_{2}$ = 0.4

Figure 4a–d show the influence of rotation parameter ${\Omega }$ on $f\left( \eta \right),\;f^{{\prime }} \left( \eta \right),\;g\left( \eta \right)\;{\text{and}}\;\theta \left( \eta \right),$ respectively, which depict that the rise the magnitude of ${\Omega }$ results in the decline in velocity field and increase in temperature filed. In physical aspect, when the values of ${\Omega }$ is larger, rotation rate gets higher than stretching rate. Therefore, higher values of ${\Omega }$ results in extra resistance for the fluid, so the velocity component behaves as decreasing function of ${\Omega }$. This study reveals that ${\Omega }$ plays an important role in the aeration of flow in y direction. It is due to the fact that higher values of ${\Omega }$ correspond to higher oscillatory motion of fluid particles. As the sheet is stretched in x-direction and due to rotation effects, the fluid flows towards y direction. Figure 4e, f depict the variable behavior of $g\left( \eta \right)\;{\text{and}}\;\theta \left( \eta \right)$ against the various values of Pr. An increase in Pr upshots a decline in temperature due to weak thermal diffusivity, therefore temperature field acts as decreasing function of Pr. Figure 5a–d exposed the effects of magnetic parameter M over the $f\left( \eta \right),\;f^{{\prime }} \left( \eta \right),\;g\left( \eta \right)\;{\text{and}}\;\theta \left( \eta \right),$ respectively. Plots show that the reduction of velocity field for the higher values of M. It is due to higher frictional forces produced by high values of M which has a trend to contract the values of velocity field. Furthermore, increase in heat transfer is observed for larger M because a certain quantity of heat is stored in the fluid when it is in the state of motion. Physically, growth in M results in stronger Lorentz force which further improves the rate of heat transfer. Whereas, Fig. 5 f display the impact of the $f\left( \eta \right)\;{\text{and}}\;\theta \left( \eta \right)$ on the radiation parameter Rd. Basically, I. e. the values of Rd provides extra heat to nanofluid which results in the rise of $\theta \left( \eta \right)$. Figure 6a–d demonstrate the impact of concentration of nanoparticles $\phi_{2}$ on the $f\left( \eta \right),\;f^{{\prime }} \left( \eta \right),\;g\left( \eta \right)\;{\text{and}}\;\theta \left( \eta \right)$, respectively. Enhancement in $f\left( \eta \right)\;and\;f^{{\prime }} \left( \eta \right)$ whereas reduction in $g\left( \eta \right)$ has been noticed for higher concentration of nanoparticles. Figure 6e, f represent the variation of $g\left( \eta \right)\;{\text{and}}\;\theta \left( \eta \right)$ against different values of $\gamma$. Higher values of $\gamma$ give increase in heat transfer rate of flow. This is because $\gamma$ depends on coefficient of heat transfer “$h_{f}$” which has larger values for greater $\gamma$. Above discussion shows that the rate of heat transfer increases with the increase in Magnetic effect,iot number and rotation rate. It is also noticed that magnetic parameter M and rotation parameter ${\Omega }$ have qualitatively same effect on velocity $g\left( \eta \right)$.

Figure 7a, b displays the numerical data in bar chart pattern for values of skin friction coefficient $C_{f} Re_{x} ^{{1/2}} = \frac{1}{{\left( {1 - \phi _{1} } \right)^{{5/2}} \left( {1 - \phi _{2} } \right)^{{5/2}} }}f^{{\prime\prime }} \left( 0 \right)$ against the $\gamma \;and\;\phi_{2} { },$ respectively. Figure 7c, d show the variation in skin friction coefficient. $C_{g} Re_{x} ^{{1/2}} = \frac{1}{{\left( {1 - \phi _{1} } \right)^{{5/2}} \left( {1 - \phi _{2} } \right)^{{5/2}} {\text{~}}}}g^{{\prime }} \left( 0 \right)$ against the different values of $M\;and\;\phi_{2}$ with bar chart representation, whereas Fig. 7e, f depict the numerical data in the form of bar charts to show the variation in heat transfer rate $Nu_{x} \text{Re} _{x} ^{{ - 1/2}} = - \frac{{k_{{hnf}} }}{{k_{f} }}\theta ^{{\prime }} \left( 0 \right)$ against $M\;and\;\Omega ,$ respectively. Table 4 depicts the computed values for skin friction and Nusselt number for all cases and scenarios. The horizontal component of skin frictions increases for scenarios I and III but reverse trend is observed against scenario V1. The scenarios II and IV have no impact on the horizontal component of skin friction. Whereas, the vertical component of skin frictions increases for scenario I, but reverse trend is observed for scenarios III and VI. The scenarios II and IV have no impact on the vertical component of skin friction. The Nusselt number is increasing for IV scenario but, opposite trend is noticed in the case of VI scenario. The scenarios I, II, III and V have no impact in the Nusselt number. The numerical calculations have been completed for two levels of convergence limits i.e. 1e-10 and 1e-12 to show the accuracy of method. Values of relative errors encountered during the computation process for all cases of each scenario are depicted in Table 5. Best value for relative errors was observed for case 1 of scenario 3 in which relative errors up to 4.1871e-13 and 4.1871e-15 are observed for 1e-10 and 1e-12 convergence limits. Table 6 shows the number of evaluations for BCs, ODEs mesh points during computational process to achieve the targeted value of accuracy. It is seen from Tables 5 and 6 that for small convergence limit, the value of relative error is improved, but at the cost of more computation in terms of ODEs and BCs evaluations. Additionally, no substantial change in relative error as well as computations of ODEs and BCs evaluations is observed by variation of scenarios and cases of system model given in Eqs. 8–12.

**Table 4:** Mathematical data for Skin friction and Nusselt number.

**Scen**	$$C_{fx} Re_{x}^{{{\raise0.7ex\hbox{$1$} \!\mathord{\left/ {\vphantom {1 2}}\right.\kern-\nulldelimiterspace} \!\lower0.7ex\hbox{$2$}}}} = \frac{{f^{{\prime \prime }} \left( 0 \right) }}{{\left( {1 - \phi_{1} } \right)^{2.5} \left( {1 - \phi_{2} } \right)^{2.5} }}$$				$$C_{fy} Re_{x}^{{{\raise0.7ex\hbox{$1$} \!\mathord{\left/ {\vphantom {1 2}}\right.\kern-\nulldelimiterspace} \!\lower0.7ex\hbox{$2$}}}} = \frac{{g^{{\prime }} \left( 0 \right)}}{{\left( {1 - \phi_{1} } \right)^{2.5} \left( {1 - \phi_{2} } \right)^{2.5} }}$$				$$Nu_{x} {\text{Re}}_{x}^{{{\raise0.7ex\hbox{${ - 1}$} \!\mathord{\left/ {\vphantom {{ - 1} 2}}\right.\kern-\nulldelimiterspace} \!\lower0.7ex\hbox{$2$}}}} = - \frac{{k_{hnf} }}{{k_{f} }}\theta^{{\prime }} \left( 0 \right)$$			
	Cases				Cases				Cases			
	I	II	III	IV	I	II	III	IV	I	II	III	IV
I	0.87437	0.88215	0.89431	0.90987	0.06598	0.13051	0.19239	0.25090	0.02303	0.02303	0.02303	0.02303
II	0.87437	0.87437	0.87437	0.87437	0.065980	0.06598	0.06598	0.06598	0.02303	0.02303	0.02303	0.02303
III	0.81524	0.87437	0.92999	1.00791	0.072317	0.065980	0.06100	0.05523	0.02304	0.02303	0.02302	0.02302
IV	0.87437	0.87437	0.874374	0.87437	0.06598	0.06598	0.06598	0.06598	0.02303	0.04546	0.06732	0.08861
V	0.87437	0.87437	0.874374	0.874374	0.06598	0.06598	0.06598	0.06598	0.02303	0.02303	0.02304	0.02304
VI	1.29661	1.24819	1.155286	1.027996	0.098245	0.094569	0.08750	0.077802	0.09179	0.06168	0.04366	0.03164

**Table 5:** Mathematical data for relative errors.

Scen	With convergence limit 1e−10				With convergence limit 1e−12			
	Cases				Cases			
	I	II	III	IV	I	II	III	IV
I	5.32098e−13	7.55295e−11	1.35957e−11	4.81301e−13	5.32098e−15	7.55295e−13	1.35957e−13	4.81301e−15
II	5.32098e−13	5.32098e−13	5.32098e−13	5.32098e−13	5.32098e−15	5.32098e−15	5.32098e−15	5.32098e−15
III	4.18713e−13	5.32098e−13	4.42368e−11	5.69376e−12	4.18713e−15	5.32098e−15	4.42368e−13	5.69376e−14
IV	5.32098e−13	5.32098e−13	5.32099e−13	5.32100e−13	5.32098e−15	5.32098e−15	5.32099e−15	5.32100e−15
V	5.32098e−13	5.32098e−13	5.32098e−13	5.32098e-13	5.32098e−15	5.32098e−15	5.32098e−−15	5.32098e-15
VI	2.12887e−12	1.84896e−12	1.39553e−12	9.23817e−13	2.12887e−14	1.84896e−14	1.39553e−14	9.23817e−15

**Table 6:** Mathematical data for ODEs, BCs evaluation and mesh points.

Scen	ODEs evaluation				BCs evaluations				Number of mesh points			
	Cases				Cases				Cases			
	I	II	III	IV	I	II	III	IV	I	II	III	IV
1	26,888	16,504	16,504	28,774	96	71	71	75	611	500	500	928
2	26,888	26,888	26,888	26,888	96	96	96	96	611	611	611	611
3	31,053	26,888	16,504	16,504	96	96	71	71	856	611	500	500
4	26,888	26,888	26,905	26,905	96	96	96	96	611	611	612	612
5	26,888	26,888	26,888	26,888	96	96	96	96	611	611	611	611
6	31,172	30,985	30,509	29,557	96	96	96	96	863	852	824	768

## Conclusions

In this study a numerical treatment for 3-D MHD flow of hybrid nanofluid over a stretchable sheet under the effects of thermal radiation has been conducted. Important findings of this research are listed as:

- Higher values of magnetic parameter causes higher frictional forces which results in decreasing of the velocity field and escalation in temperature field.
- Decline in the velocity field is noticed for the increasing values of rotation parameter, while, the reverse performance is experienced for the case of temperature field.
- Enhancement in velocity and temperature fields is perceived against the large values of Biot number and concentration of nanoparticles $\phi_{2}$.
- For greater values of Prandtl number the velocity field $f\left( \eta \right)$ increases, while temperature filed reduces.
- Values of skin friction $$C_{f} Re_{x}^{{{\raise0.7ex\hbox{$1$} \!\mathord{\left/ {\vphantom {1 2}}\right.\kern-\nulldelimiterspace} \!\lower0.7ex\hbox{$2$}}}}$$ boost with increasing M and decline with the greater concentration $$\phi_{2}$$.
- Skin friction $$C_{g} Re_{x}^{{{\raise0.7ex\hbox{$1$} \!\mathord{\left/ {\vphantom {1 2}}\right.\kern-\nulldelimiterspace} \!\lower0.7ex\hbox{$2$}}}}$$ decreases for larger values of magnetic parameter and rises for higher values of rotation parameter $$\Omega$$.
- Nusselt Number rises with the increase in Biot number, while opposite behavior is observed for $\phi_{2}$.

In future one may explore the different characteristics of 3-D MHD flow of hybrid nanofluid with thermal radiation features through modern and advanced numerical computing skills based of artificial intelligence^60–66^.

## Symbols

*T*: Temperature
*u, v, w*: Components of velocity
*f, g*: Dimensionless velocities
$$\theta$$: Dimensionless temperature
h: Heat transfer coefficient
s: Shape factor
*Nu*: Nusselt number
*Re*: Reynolds number
*k*: Thermal conductivity
*C*_*P*_: Specific heat
*Rd*: Radiation parameter

## Subscripts

*nf*: Nanofluid
*hnf*: Hybrid nanofluid

## Greek letters

$$\rho$$: Density
$$\mu$$: Dynamic viscosity
$$\nu$$: Kinematic viscosity
$$\eta$$: Transformed coordinate
$$\omega$$: Angular velocity
$\phi$: Nano particle volume fraction
$\Omega$: Transformed angular velocity
$$\sigma$$: Electrical conductivity
$$\gamma$$: Biot number

## Abbreviations

CNTs: Carbon nanotubes
ODEs: Ordinary differential equations
PDEs: Partial differential equations
MCHS: Micro channel heat sink
MWCNTs: Multi wall CNTs
MWCNTs: Multi-wall CNTs
MHD: Magnetohydrodynamics
